# Association between miR-365 polymorphism and ischemic stroke in a Chinese population

**DOI:** 10.3389/fneur.2023.1260230

**Published:** 2023-09-27

**Authors:** Yin-Hua Weng, Wen-Tao Yu, Yan-Ping Luo, Chao Liu, Xi-Xi Gu, Huo-Ying Chen, Hong-Bo Liu

**Affiliations:** ^1^Department of Laboratory Medicine, The Second Affiliated Hospital of Guilin Medical University, Guilin, China; ^2^College of Medical Laboratory Science, Guilin Medical University, Guilin, China; ^3^School of Clinical Medicine, Guilin Medical University, Guilin, China; ^4^Guangxi Health Commission Key Laboratory of Glucose and Lipid Metabolism Disorders, Guangxi Key Laboratory of Metabolic Reprogramming and Intelligent Medical Engineering for Chronic Diseases, The Second Affiliated Hospital of Guilin Medical University, Guilin, China

**Keywords:** ischemic stroke, polymorphism, miR-365, genotype, risk, miRNA

## Abstract

**Background:**

Ischemic stroke (IS) represents a major cause of morbidity and mortality across the globe. The aberrant expression of miR-365 has been found to be implicated in a wide array of human diseases, including atherosclerosis and cancer. Studies on single-nucleotide polymorphisms (SNPs) in miRNA genes can help gain insight into the susceptibility to the condition. This study aimed to examine the relationship between miR-365 SNPs and the risk of IS.

**Methods:**

The study recruited 215 IS patients and 220 controls. The SNPscans genotyping was employed to genotype three polymorphic loci (rs121224, rs30230, and rs178553) of miR-365. The relative expression of miR-365 in peripheral blood mononuclear cells of the patients and controls was determined by using real-time quantitative PCR.

**Results:**

The miR-365 rs30230 polymorphism exhibited a significant association with the risk of developing IS (TC vs. CC: adjusted OR = 0.55, 95% CI: 0.33-0.92, *P* = 0.022; TT vs. CC: adjusted OR = 0.34, 95% CI: 0.14–0.85, *P* = 0.021; TC +TT vs. CC: adjusted OR = 0.51, 95% CI: 0.31–0.83, *P* = 0.007; T vs. C: adjusted OR = 0.57, 95% CI: 0.39–0.83, *P* = 0.004). Haplotype analysis revealed that the C-T-G haplotype was associated with a decreased risk of IS (OR = 0.68, 95% CI: 0.46–1.00, *P* = 0.047). Furthermore, miR-365 expression was significantly higher in IS patients than in controls (*P* < 0.001). Interestingly, patients with rs30230 TC or TT genotypes had lower miR-365 levels compared to their counterparts with CC genotypes (*P* < 0.001).

**Conclusions:**

The miR-365 rs30230 polymorphism might bear an association with IS susceptibility in the Chinese population, and the rs30230 TC/TT genotype might be a protective factor against IS.

## 1. Introduction

Stroke represents a highly prevalent cardiovascular and cerebrovascular disease globally and is characterized by a significantly higher morbidity and mortality. With annual deaths standing at 50,000, it ranks as the second most lethal disease worldwide (1, 2). The rising incidence and the sheer patient numbers impose an immense burden on medical resources and society at large (3). Ischemic stroke (IS) is etiologically multifactorial and is linked to various risk factors, including age, gender, smoking, alcohol abuse, diabetes, hyperlipidemia, and hyperhomocysteinemia (4, 5). In addition to those well-established factors, genetic factors have increasingly been on the radar of researchers due to their association with ischemic stroke. Extensive evidence now supports the involvement of genetic variants in the susceptibility to IS (6–9). MicroRNAs, a class of endogenous small non-coding RNAs, are highly conservative across species and typically consist of 18–22 nucleotides. They primarily regulate gene expression *in vivo* by binding complementarily to the 3′UTR of target mRNAs, thereby modulating mRNA translation *via* repressing or degrading their target RNAs at the transcriptional level (10, 11). Currently, mounting evidence suggests that a wide array of microRNAs is implicated in the progression of IS. For instance, miR-497 was reported to be closely associated with unfavorable prognosis of IS patients (12), and the overexpression of miR-181c was shown to exacerbate cerebral damage in patients with acute ischemic stroke (13). As a member of the microRNA family, miR-365 was reportedly linked to some mechanistic processes, such as IL-6 regulation and vascular smooth muscle cell proliferation, both being involved in the pathogenesis of atherosclerosis (14, 15). Atherosclerosis is intimately correlated with cardiovascular and cerebrovascular diseases, including coronary heart disease and IS. Moreover, miR-365 expression was found to be aberrant in a rat model of middle cerebral artery occlusion, and it was shown to take part in the development, progression, and prognosis of stroke *via* multiple mechanisms (16, 17). On the basis of these findings, we are led to speculate that miR-365 might serve as a biomarker for the early diagnosis and prognostic assessment of IS and a target for the early intervention of IS.

Single-nucleotide mutations in miRNAs may impact miRNAs expression, thereby affecting the susceptibility to diseases and prognosis (18, 19). Of note, a study conducted in a northern Chinese population demonstrated that polymorphism at the rs121224 locus of miR-365 gene was associated with both the susceptibility to and prognosis of intestinal gastric cancer (20). Based on this background, we were further led to theorize that miR-365 might be involved in the pathogenesis of human IS. We further postulated that miR-365 SNPs might, in some way, bear association with the susceptibility to IS. In this study, we looked at the genetic susceptibility conferred by three miR-365 SNPs (rs121224, rs30230, and rs178553) and assessed their correlation with miR-365 expression, in an attempt to find IS-susceptibility-related genes and their corresponding genotypes. Understanding such associations will help us identify high-risk populations for early screening, prevention, and intervention of IS.

## 2. Materials and methods

### 2.1. Study population

The study population consisted of 215 patients diagnosed with IS who were admitted to the Affiliated Hospital of Guilin Medical University, Guangxi, China, between June 2020 and June 2021. A total of 220 sex- and age-controlled subjects who received physical checkups at the same hospital were included and served as controls. The diagnosis of IS was based on the diagnostic criteria contained in the Chinese Guidelines for the Diagnosis and Treatment of Acute Ischemic Stroke (21), formulated by the Chinese Academy of Neurology. Study subjects were excluded if they had a history of cerebral hemorrhage, intracranial infections, malignancies in any system, hematological diseases, autoimmune diseases, or infectious diseases. Clinical data were collected, including age, gender, diabetes status, and lipid profile [total cholesterol (TC), triglyceride (TG), high-density lipoprotein cholesterol (HDL-C), and low-density lipoprotein cholesterol (LDL-C)]. Moreover, it is worth noting that all study subjects were unrelated to the Guangxi population to ensure genetic diversity within the sample.

### 2.2. Ethics

The study protocol received ethics approval from the Ethics Review Committee of the Affiliated Hospital of Guilin Medical University, Guilin, China. Prior to their inclusion, all participants provided written informed consent with their signatures, indicating their willingness to take part in the study.

### 2.3. SNPs selection

Three SNPs (rs121224, rs30230, and rs178553) were selected for further analysis according to the following criteria. The criteria included those as follows: (1) The frequency of minor alleles in the Chinese Han population was >5%; (2) SNPs located in genetically important functional regions, such as promoter regions; and (3) SNPs that had been reported in the literature were selected in combination with literature references in PubMed.

### 2.4. DNA extraction and genotyping

Approximately 3–5 ml of blood samples of the subjects were collected, after an overnight fast, into EDTA-K2 anticoagulant tubes. Genomic DNA was subsequently extracted by utilizing a DNA extraction kit (DP318, Qiagen, China). Detection was performed according to instructions of the SNPscan^TM^ multiplex SNP typing kit, and the primer data are listed in Table 1. PCR products were purified by employing shrimp alkalase (SAP) (from Promega) and exonuclease I (EXOI) (from Epicenter) and sequenced by using ABI's BigDye3.1 kit and on the ABI3730XL system (PE Applied Biosystems, Foster City, CA, USA) sequencer where raw data were collected and analyzed with the software GeneMapper 4.1 (Applied Biosystems, USA). Moreover, an additional 3–5 ml of venous blood was drawn from the subjects and centrifuged to separate the serum for lipid profiling.

**Table 1:** Primer sequences.

**SNP**	**Forward primer (5^′^-3^′^)**	**Reverse primer (5^′^-3^′^)**
rs121224	CTTCTGGGCACTTCCTGGT	CGGGAACGTGGCAAGAAC
rs30230	GTAGTAAGCTCCTCCTCTGGTGTCA	GCTGAGTGGTTATCGCTGGTG
rs178553	AATAGAAGGAGCCTCAGAATCCAC	TAGGTGGGGGAGCCCATT

### 2.5. Quantitative PCR of miR-365

Total RNA was extracted from peripheral blood single nuclei by using the TRIzol method, and the extracts were measured at 260 nm and 280 nm on a UV spectrophotometer to assess the purity of RNA samples. The RNA was then reverse-transcribed into cDNA by using a commercial kit (MonScript^TM^ miRNA First Strand cDNA Synthesis Kit, REF: MR05301), and cDNA was amplified with an amplification kit (MonAmp^TM^ SYBR^®^ Green qPCR Mix with None/Low/High ROX, REF: MQ10101/MQ10201/MQ10301). The forward primer was 5′-TAATGCCCCTAAAAATCC-3′. To compare the differences in miR-365 expression of the peripheral blood monocytes between IS patients and controls, relative quantitative analysis was conducted by using the 2^−Δ*ΔCt*^ method. Additionally, the impact of polymorphisms on gene expression was analyzed to assess their association with miR-365 expression.

### 2.6. Statistical analysis

Statistical analysis was performed using IBM SPSS 25.0 (SPSS Inc., Chicago, IL, USA). The continuous data were expressed as mean ± standard deviation (SD) and compared using Student's *t*-test. The χ^2^ test was performed to assess the Hardy–Weinberg equilibrium (HWE) and analyze categorical data, such as gender and status of diabetes mellitus. After adjustment for age, sex, diabetes status, TC, TG, HDL-C, and LDL-C, logistic regression was employed to evaluate the correlation between miR-365 polymorphisms and IS susceptibility by odds ratio (OR), 95% confidence interval (CI), and *P*-value. Linkage disequilibrium (LD) and haplotype analysis were performed by using SHEsis software (http://analysis.bio-x.cn/myAnalysis.php). A *P* < 0.05 was considered to be statistically significant.

## 3. Results

### 3.1. The study population characteristics

The demographic and clinical features of IS patients and healthy controls are given in Table 2. No significant differences were observed between patients and controls in terms of gender, age, and TG levels. However, IS patients exhibited significantly higher levels of TC and LDL-C and a lower level of HDL-C compared to controls (all *P* < 0.05).

**Table 2:** Clinical data of the study population.

**Variables**	**Controls, *n* = 220**	**IS patients, *n* = 215**	***P-*value**
Age, years (Mean ± SD)	63.34 ± 0.44	64.55 ± 0.73	0.154
Gender (M/F)	130/90	121/94	0.553
Diabetes mellitus (%)	26 (11.8)	42 (19.5)	< 0.001
TC (mmol/L)	4.83 ± 0.56	3.99 ± 0.07	< 0.001
TG (mmol/L)	1.61 ± 0.07	1.67 ± 0.07	0.495
HDL-C (mmol/L)	1.41 ± 0.03	1.02 ± 0.02	< 0.001
LDL-C (mmol/L)	2.63 ± 0.06	2.82 ± 0.05	0.020

IS, ischemic stroke; SD, standard deviation; TG, triglyceride; TC, total cholesterol; HDL, high-density lipoprotein; LDL, low-density lipoprotein.

### 3.2. Association of rs121224, rs30230, and rs178553 polymorphisms with the risk of IS

The impact of the miR-365 SNPs on the risk of IS is presented in Table 3. Genotypic distribution in both patients and controls was consistent with HWE (*P* > 0.05). Rs30230 TC, TT, and TC+TT genotypes were associated with a reduced risk of IS with corrected ORs of 0.55, 0.34, and 0.51, respectively (TC vs. CC: 95% CI, 0.33–0.92, *P* = 0.022; TT vs. CC: 95% CI, 0.14–0.85, *P* = 0.021; TC + TT vs. CC: 95% CI, 0.31–0.83, *P* = 0.007). Similarly, subjects carrying the T allele had a lower risk for IS than the carriers of the C allele (OR = 0.57, 95% CI, 0.39–0.83, *P* = 0.004), while rs121224 and rs178553 were not significantly associated with IS risk.

**Table 3:** Association between miR-365 polymorphisms and risk of IS.

**Polymorphisms**	**Controls, *n* = 220**	**IS patients, *n* = 215**	**OR (95% CI)^†^**	***P*−*value*^†^**
**rs121224**				
CC	66 (30.0)	70 (32.6)	1.00 (ref)	
GC	106 (48.2)	98 (45.6)	1.04 (0.60–1.82)	0.882
GG	48 (21.8)	47 (21.8)	1.00 (0.54–1.96)	0.989
C	238 (54.1)	238 (55.3)	1.00 (ref)	
G	202 (45.9)	192 (44.7)	1.00 (0.71–1.40)	0.968
**Dominant model**				
GG	48 (21.8)	47 (21.8)	1.00 (ref)	
CC+CG	172 (78.2)	168 (78.2)	1.03 (0.57–1.87)	0.921
**Recessive model**				
GG+CG	154 (70.0)	145 (67.4)	1.00 (ref)	
CC	66 (30.0)	70 (32.6)	0.97 (0.58–1.63)	0.917
HWE (*P-*value)	0.658	0.254		
**rs30230**				
CC	104 (47.3)	127 (59.1)	1.00 (ref)	
TC	95 (43.2)	73 (34.0)	0.55 (0.33–0.92)	0.022
TT	21 (9.5)	15 (6.9)	0.34 (0.14–0.85)	0.021
C	303 (68.9)	327 (76.0)	1.00 (ref)	
T	137 (31.1)	103 (24.0)	0.57 (0.39–0.83)	0.004
**Dominant model**				
CC	104 (47.3)	127 (59.1)	1.00 (ref)	
TT+TC	116 (52.7)	88 (40.9)	0.51 (0.31–0.83)	0.007
**Recessive model**				
CC+TC	199 (90.5)	200 (93.1)	1.00 (ref)	
TT	21 (9.5)	15 (6.9)	0.45 (0.19–1.08)	0.072
HWE (*P-*value)	0.918	0.319		
**rs178553**				
AA	47 (21.3)	47 (21.8)	1.00 (ref)	
AG	106 (48.2)	98 (45.6)	1.04 (0.55–1.96)	0.916
GG	67 (30.5)	70 (32.6)	0.95 (0.48–1.88)	0.882
A	200 (45.5)	192 (44.7)	1.00 (ref)	
G	240 (54.5)	238 (55.3)	0.97 (0.69–1.37)	0.873
**Dominant model**				
GG	67 (30.5)	70 (32.6)	1.00 (ref)	
AA+AG	153 (69.5)	145 (67.4)	1.08 (0.64–1.81)	0.776
**Recessive model**				
GG+AG	173 (78.7)	168 (78.2)	1.00 (ref)	
AA	47 (21.3)	47 (21.8)	1.00 (0.55–1.82)	0.999
HWE (*P-*value)	0.674	0.254		

OR, odds ratio; CI, confidence interval; HWE, Hardy–Weinberg equilibrium;^†^adjusted by age, gender, diabetes mellitus, TG, TC, LDL-C, and HDL-C.

### 3.3. Haplotype analysis of rs121224, rs30230, and rs178553 polymorphisms and risk of IS

**Figure 1:**
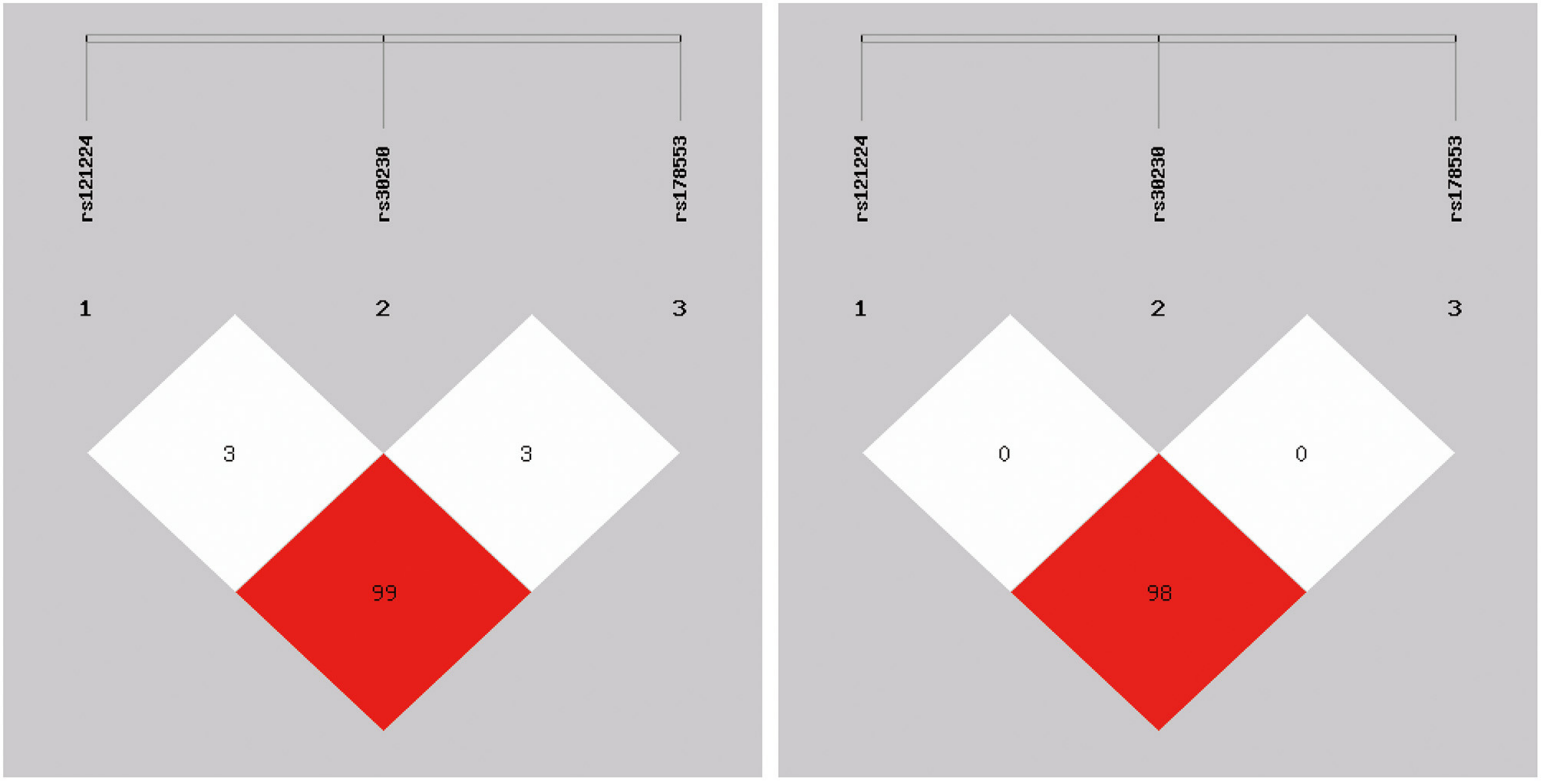
Linkage disequilibrium test of the three polymorphisms. The rs121224 polymorphism was in linkage disequilibrium (LD) with rs178553(D′ = 0.99, *r*^2^ = 0.98).

To further evaluate the relationship between miR-365 polymorphism and IS risk, we performed haplotype analysis. The results of the analysis showed that there was linkage disequilibrium among the three sites (Figure 1), with rs121224 and rs178773 exhibiting strong linkage disequilibrium (*D*′ = 0.99, *r*^2^ = 0.98). As shown in Table 4, four possible haplotypes were listed, and the C-T-G haplotype had a tendency to reduce IS susceptibility (OR = 0.68, 95% CI, 0.46–1.00, *P* = 0.047).

**Table 4:** Haplotype analysis of miR-365 polymorphisms with IS risk.

**rs121224**	**rs30230**	**rs178553**	**Controls (%)**	**IS (%)**	**OR (95%CI)**	***P-*value**
C	C	G	164 (37.3)	185 (43.1)	1.27 (0.97–1.67)	0.083
C	T	G	74 (16.7)	52 (12.0)	0.68 (0.46–1.00)	0.047
G	C	A	137 (31.1)	142 (33.0)	1.09 (0.82–1.45)	0.561
G	T	A	63 (14.3)	49 (11.5)	0.77 (0.52–1.15)	0.207

OR, odds ratio; CI, confidence interval; only frequency >1% is presented.

### 3.4. Combined analysis

Given that the SNP association analysis revealed a protective effect of the rs30230 TC/TT genotype on IS risk, we further investigated whether rs121224–rs30230 in combination with rs178553–rs30230 exerted a synergic effect on IS risk. However, as indicated in Table 5, no significant correlation was observed between the combination of the two genotypes and IS risk (*P* > 0.05).

**Table 5:** Synergic effects of miR-365 SNPs on the risk of IS.

**Combined genotypes**	**Controls (%)**	**IS (%)**	**OR (95% CI)**	***P-*value**
**rs121224-rs30230**				
rs121224GG+rs30230CC	24 (10.9)	25 (11.6)	1.00	
rs121224GG+rs30230TC/TT	24 (10.9)	22 (10.2)	0.88 (0.39–1.97)	0.756
rs121224CC/CG+rs30230CC	80 (36.4)	102 (47.5)	1.22 (0.65–2.30)	0.531
rs121224CC/CG+rs30230TC/TT	92 (41.8)	66 (30.7)	0.69 (0.36–1.31)	0.256
**rs178553-rs30230**				
rs178553GG+rs30230CC	34 (15.5)	39 (18.2)	1.00	
rs178553GG+rs30230TC/TT	33 (15.0)	31 (14.4)	0.82 (0.42–1.60)	0.560
rs178553AG/AA+rs30230CC	70 (31.8)	88 (40.9)	1.10 (0.63–1.91)	0.747
rs178553AG/AA+rs30230TC/TT	83 (37.7)	57 (26.5)	0.60 (0.34–1.06)	0.078

SNP, single-nucleotide polymorphism; IS, ischemic stroke; OR, odds ratio; CI, confidence interval.

### 3.5. Multiple logistic regression analysis

In this study, it was observed that TC levels were lower in IS patients than in controls, and there was no significant difference in the TG level between the two groups. This finding could be attributed to the prior medication on the part of the patients. In light of this, in the multiple logistic regression analysis, TG and TC were excluded, and only the effects of HDL-C, LDL-C, and rs30230 TC/TT were analyzed. The results, as presented in Table 6, demonstrated that the combination of rs30230 TC/TT genotype and lipid profile had an impact on the risk of IS. The specific data were as follows: HDL-C (OR = 0.01; 95% CI, 0.01–0.03), LDL-C (OR = 1.38; 95% CI, 1.05–1.82), and rs30230 TC/TT (OR = 0.47; 95% CI, 0.30–0.75) (all *P* < 0.05).

**Table 6:** Logistic regression analysis for identifying risk factors of IS.

**Variables**	**B**	**S. E**	***P-*value**	**OR (95%CI)**
HDL-C	−4.403	0.470	< 0.001	0.01 (0.01–0.03)
LDL-C	0.323	0.142	0.023	1.38 (1.05–1.82)
rs30230TC/TT	−0.752	0.235	0.001	0.47 (0.30–0.75)

OR, odds ratio; CI, confidence interval; HDL, high-density lipoprotein; LDL, low-density lipoprotein.

### 3.6. Association between rs121224, rs30230, and rs178553 polymorphisms and serum lipid levels in IS patients

We further explored the potential association between rs121224, rs30230, and rs178553 polymorphisms and serum levels of TG, TC, HDL-C, and LDL-C in the IS group. The results, as given in Table 7, did not reveal any significant relationship between these three polymorphisms and the aforementioned lipid parameters in the IS group (*P* > 0.05).

**Table 7:** Stratification of miR-365 SNPs and clinical characteristics of IS.

**Polymorphisms**	***n***	**TC (mmol/L)**	**TG (mmol/L)**	**HDL-C (mmol/L)**	**LDL-C (mmol/L)**
**rs121224**				
GG	47	3.85 ± 0.12	1.53 ± 0.13	1.03 ± 0.35	2.77 ± 0.10
CC+CG	168	4.04 ± 0.08	1.71 ± 0.08	1.03 ± 0.02	2.83 ± 0.06
*t*-values		−1.10	−1.10	0.05	−0.54
*P-*values		0.272	0.213	0.958	0.592
**rs30230**				
CC	127	3.95 ± 0.09	1.69 ± 0.89	1.03 ± 0.02	2.84 ± 0.07
TT+TC	88	4.05 ± 0.11	1.65 ± 0.11	1.01 ± 0.02	2.79 ± 0.08
*t*-values		−0.69	0.29	1.12	0.52
*P-*values		0.488	0.774	0.265	0.605
**rs178553**				
GG	70	3.87 ± 0.13	1.76 ± 0.13	1.03 ± 0.03	2.80 ± 0.10
AA+AG	145	4.05 ± 0.08	1.63 ± 0.08	1.03 ± 0.02	2.83 ± 0.06
*t*-values		−1.20	0.87	0.02	−0.29
*P-*values		0.233	0.383	0.982	0.775

TG, triglyceride; TC, total cholesterol; HDL, high-density lipoprotein; LDL, low-density lipoprotein.

### 3.7. Genotype distribution of rs30230 polymorphism in various populations

We further compared the distribution of the rs30230 polymorphism across different populations with respect to the significance of its polymorphism in the etiology of IS. The results, as given in Table 8, showed that the genotype distribution of rs30230 in this study was statistically significant (*P* < 0.05) when compared with the HapMap-CDX and HapMap-JPT populations in Asians. Statistically significant differences (*P* < 0.05) also existed in the distribution when compared with the European HapMap-CUE, HapMap-FIN, and HapMap-GBR and also the American HapMap-PEL, the South Asian HapMap-BEB, HapMap-PJL, and African populations.

**Table 8:** Genotype distribution of the rs30230 polymorphism in various populations.

**Populations**	***n***	**Genotype frequency (%)**			***P***	**Ethnic**
		**CC**	**TC**	**TT**		
Our date	220	104 (47.3)	95 (43.2)	21 (9.5)	-	Asian
HapMap-CHB	103	57 (55.3)	42 (40.8)	4 (3.9)	0.141	Asian
HapMap-CDX^*^	93	56 (60.2)	26 (28.0)	11 (11.8)	**0.041**	Asian
HapMap-JPT^*^	104	63 (60.6)	37 (35.6)	4 (3.8)	**0.041**	Asian
HapMap-KHV	99	59 (59.6)	36 (36.4)	4 (4.0)	0.066	Asian
HapMap-YRI^*^	108	107 (99.1)	1 (0.9)	-	**< 0.001**	African
HapMap-LWK^*^	99	94 (94.9)	5 (5.1)	-	**< 0.001**	African
HapMap-ESN^*^	99	98 (99.0)	1 (1.0)	-	**< 0.001**	African
HapMap-ASW^*^	61	52 (85.2)	7 (11.5)	2 (3.3)	**< 0.001**	African
HapMap-ACB^*^	96	90 (93.8)	6 (6.2)	-	**< 0.001**	African
HapMap-GWD^*^	113	111 (98.2)	2 (1.8)	-	**< 0.001**	African
HapMap-MSL^*^	85	84 (98.8)	1 (1.2)	-	**< 0.001**	African
HapMap-CEU^*^	99	38 (38.4)	38 (38.4)	23 (23.2)	**0.004**	European
HapMap-FIN^*^	99	33 (33.3)	50 (50.5)	16 (16.2)	**0.039**	European
HapMap-TSI	107	53 (49.5)	42 (39.3)	12 (11.2)	0.763	European
HapMap-GBR^*^	91	32 (35.2)	35 (38.5)	24 (26.4)	**0.001**	European
HapMap-IBS	107	57 (53.3)	44 (41.1)	6 (5.6)	0.377	European
HapMap-CLM	94	37 (39.4)	41 (43.6)	16 (17.0)	0.132	American
HapMap-MXL	64	23 (35.9)	31 (48.4)	10 (15.6)	0.182	American
HapMap-PEL^*^	85	15 (17.6)	41 (48.2)	29 (34.1)	**< 0.001**	American
HapMap-PUR	104	46 (44.2)	45 (43.3)	13 (12.5)	0.695	American
HapMap-BEB^*^	86	54 (62.8)	29 (33.7)	3 (3.5)	**0.028**	South Asian
HapMap-GIH	103	55 (53.4)	42 (40.8)	6 (5.8)	0.408	South Asian
HapMap-ITU	102	62 (60.8)	32 (31.4)	8 (7.8)	0.076	South Asian
HapMap-PJL^*^	96	51 (53.1)	28 (29.2)	17 (17.7)	**0.023**	South Asian
HapMap-STU	102	42 (41.2)	48 (47.1)	12 (11.8)	0.565	South Asian

CHB, Han Chinese in Beijing, China; CDX, Chinese Dai in Xishuangbanna, China; JPT, Japanese in Tokyo, Japan; KHV, Kinh in Ho Chi Minh City, Vietnam; YRI, Yoruba in Ibadan, Nigeria; LWK, Luhya in Webuye, Kenya; ESN, Esan in Nigeria; ASW, Americans of African Ancestry in SW USA; ACB, African Caribbeans in Barbados; GWD, Gambian in Western Divisions in the Gambia; MSL, Mende in Sierra Leone; CEU, Utah Residents (CEPH) with Northern and Western European Ancestry; FIN, Finnish in Finland; TSI, Toscani in Italia; GBR, British in England and Scotland; IBS, Iberian Population in Spain; CLM, Colombians from Medellin, Colombia; MXL, Mexican Ancestry from Los Angeles USA; PEL, Peruvians from Lima, Peru; PUR, Puerto Ricans from Puerto Rico; BEB, Bengali from Bangladesh; GIH, Gujarati Indian from Houston, Texas; ITU, Indian Telugu from the UK; PJL, Punjabi from Lahore, Pakistan; STU, Sri Lankan Tamil from the UK.

^*^P < 0.05. Both ^*^ and bold values denote *p*-values < 0.05; In this manner, the significant differences are easy being found.

### 3.8. Genotype of rs30230 polymorphism is associated with miR-365 expression

**Figure 2:**
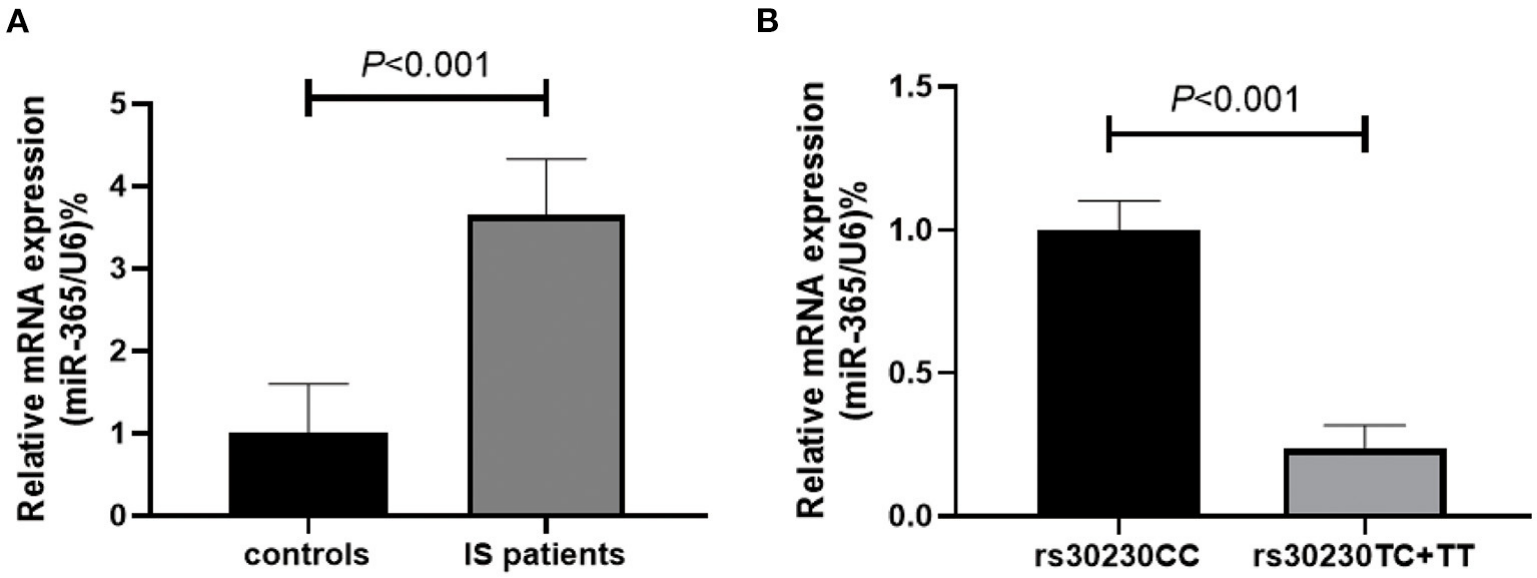
**(A)** Relative expression of the miR-365 in IS patients (*n* = 30) and controls (*n* = 30) in peripheral blood mononuclear cells. **(B)** The association between rs30230 polymorphism and the expression of miR-365 in IS patients. Patients carrying rs30230 TC or TT genotype (*n* = 20) had a significantly lower level of miR-365 as compared with the carriers of rs302030 genotype (*n* = 10).

To investigate the potential influence of rs30230 gene polymorphism on the expression level of miR-365 in IS patients, 30 IS patients and 30 controls were randomly selected for miR-365 testing of whole blood, and the control group was used for comparison. The results revealed a significantly higher expression of miR-365 in IS patients (*P* < 0.001, Figure 2A). Furthermore, we further determined the miR-365 expression in the IS group, focusing on patients carrying different genotypes. Specifically, we detected miR-365 expression in 10 patients for each genotype. The findings demonstrated that individuals with the rs30230 TC+TT genotype had lower levels of miR-365 expression compared to those having the rs30230 CC genotype (*P* < 0.001, Figure 2B).

## 4. Discussion

This study aimed to investigate the association between the development of IS and three SNPs, namely rs121224, rs30230, and rs178553, in the miR-365 gene. The findings revealed a significant correlation between TC and TT genotypes of the rs30230 polymorphism and a reduced risk of IS. Moreover, our analysis demonstrated that IS patients carrying the TC and TT genotypes of rs30230 had a lower miR-365 expression level compared to their counterparts with the CC genotype. These results suggest that a correlation might exist between the miR-365 rs30230 polymorphism and susceptibility to IS in the Chinese population.

As a member of the microRNAs family, miR-365 plays a vital role in a multitude of biological events, such as chondrocyte development (22), pathogenesis of cancer (23), and atherosclerosis (14). Recently, growing evidence has suggested that miR-365 is implicated in the development of neurological diseases. It was reportedly associated with morphine tolerance and nociceptive behavior (24, 25), and its aberrant expression was observed in the spinal cord of amyotrophic lateral sclerosis rats and in the hippocampus of epileptic rats (26, 27). Of note, the upregulated miR-365 expression led to augmented neurological deficits and brain infarct size by reducing the level of PAX6 expression and the number of newly mature neurons derived from astrocytes in the ischemic striatum (16). The dysregulated expression of miR-365 was also found to be connected to oxidative damage in rat ischemic brain (17). A study using the atherosclerotic model showed that miR-365 enhanced ox-LDL-induced endothelial apoptosis by regulating Bcl-2 expression (28). Furthermore, miR-365 was upregulated in extracellular vesicles isolated from cholesterol-loaded macrophages (29). In another study, multiple target genes related to the regulation of miR-365-2-5p were exhibited to be associated with functions related to cholesterol metabolism (30). These were all promotors to the development of atherosclerosis. Moreover, atherosclerosis is generally believed to be a crucial pathological process underlying cardiovascular diseases. Therefore, it is only natural to theorize that miR-365 might play a crucial part in the development and progression of IS and can be used as a therapeutic target for the treatment of the condition.

Most previous studies investigating the association between genetic polymorphisms and susceptibility to IS have primarily focused on protein-coding genes (31, 32). However, in recent years, research efforts have been increasingly directed at the exploration of the relationship between miRNA-associated polymorphisms and the risk of IS (33). The miRNA gene variants may lead to abnormalities in their expression and may even bear an association with the susceptibility to a certain disease (34, 35). With the advancements in genome-wide research, a growing number of studies have revealed associations between SNPs in miRNA regions and IS. For instance, Han et al. identified an association between the miR-181b gene rs322931 CT/TT genotype and IS susceptibility (36). On the other hand, Li et al. found that the miR-155 expression was elevated in whole-blood samples from IS patients but failed to observe a significant association between miR-155 gene rs767649 polymorphism and miR-155 expression (37). These findings suggest that SNPs may play a crucial role in the etiology of IS. On the strength of the prior results, we were led to hypothesize that SNPs in miR-365 are associated with the risk of IS. In our experimental investigations, we confirmed this hypothesis and found a correlation between miR-365 polymorphism and IS risk. To the best of our knowledge, this is the first study to reveal the relationship between rs30230 and IS. Our results indicated that the rs30230 TC/TT genotype was strongly associated with a reduced risk of IS. Additionally, our findings suggested that the T allele serves as a protective genotype against IS, and the C-T-G haplotype is associated with a decreased risk of IS. However, SNPs rs121224 and rs178553 did not show a significant association with IS susceptibility. Furthermore, we did not find a synergic protective effect of rs121224–rs30230 in combination with rs178553-rs30230 on IS risk possibly due to the limited loci covered by the study, and, presumably, rs121224 may work jointly with other loci to achieve a combined effect, which warrants further study. Our multiple logistic regression analysis identified the rs30230 TC/TT genotype as an independent protective factor for IS. An altered lipid profile is a well-established risk factor for the formation of atherosclerotic plaques. Atherosclerotic plaque formation can lead to blood flow blockage, ischemia, and hypoxia, thereby contributing to severe cardiovascular and cerebrovascular diseases, such as IS and coronary heart disease (38). We then further investigated the relationship between miR-365 SNPs and lipid levels in IS patients. Unfortunately, we failed to observe any significant association. One possible explanation is that the number of IS subjects in our study was relatively small compared to the overall study population, and the observed results might be fortuitous. Another reason might be the potential interaction between these loci and environmental factors during the progression of IS. As shown in Table 8, the genotypes of SNPs vary substantially across regions and races, and both genetic and environmental factors are likely to synergistically influence the susceptibility to disease, which needs further evaluation and exploration.

We then assessed the relative expression of miR-365 in PBMCs in IS patients and controls and found that miR-365 expression was significantly upregulated in IS patients as compared to controls. This result further substantiated our conjecture regarding the potential involvement of miR-365 in IS development. Premised on this finding, we assumed that rs30230 polymorphism might influence miR-365 expression and, consequently, contribute to the progression of IS. To test this assumption, we examined the association between different genotypes of rs30230 in IS patients and miR-365 expression levels. Remarkably, we observed a significantly lower miR-365 level in patients carrying the rs30230 TC or TT genotypes relative to those with the rs30230 CC genotype. This finding lends support to the notion that rs30230 polymorphism in miR-365 is associated with gene expression. These findings collectively suggest that miR-365 and its rs30230 polymorphism may play a crucial role in the pathogenesis of IS.

The current study highlights the significant role of miR-365 polymorphisms in the etiology of IS. However, certain limitations should be acknowledged. First, our analysis was based on a limited set of clinical data, and the sample size for testing was relatively small. Therefore, the association between miR-365 and IS populations should be further confirmed in large-sized samples. Second, it is important to recognize that there might be a potential selection bias since all participants were recruited from a single setting. This limits the extrapolation of our findings to populations with different genetic and environmental backgrounds. Third, IS was known to result from complex interactions between genetic and environmental factors, and genetic polymorphisms may vary with populations. Therefore, caution should be exercised when extrapolating our results to other ethnic or racial populations with varying genetic backgrounds and environmental contexts. To overcome these limitations, future research efforts should incorporate more functional experiments and animal studies for further validation. By doing so, we can gain a deeper insight into the specific role played by these SNPs in the development of IS. Such approaches will help strengthen the validity and reliability of our findings and further enhance our understanding of the link between SNPs and IS.

## 5. Conclusion

In conclusion, our study provided preliminary evidence that the miR-365 rs30230 polymorphism is associated with susceptibility to IS. Specifically, we observed a protective role of the T allele, TC genotype, TT genotype, and dominant model in the development of IS. These findings underscore the potential of rs30230 as a biomarker for predicting the development and progression of IS.

## Data availability statement

The original contributions presented in the study are publicly available. This data can be found here: European Molecular Biology Laboratory's European Bioinformatics Institute (EMBL-EBI), European Variation Archive (EVA), https://www.ebi.ac.uk/eva/, PRJEB65578.

## Ethics statement

The studies involving human participants were reviewed and approved by the Review Board of the Affiliated Hospital of Guilin Medical University (2022YJSLL-80). Written informed consent to participate in this study was provided by the patients/participants.

## Author contributions

Y-HW: Methodology, Writing—original draft. W-TY: Methodology, Writing—original draft. Y-PL: Formal analysis, Writing—review and editing, Data curation. CL: Data curation, Formal analysis, Writing—review and editing. X-XG: Data curation, Formal analysis, Writing—review and editing. H-YC: Conceptualization, Writing—review and editing. H-BL: Conceptualization, Writing—review and editing. All authors have read and approved the final manuscript.
